# Transcriptome RNA Sequencing Data Set of Gene Expression in Moraxella catarrhalis On- and Off-Phase Variants of the Type III DNA Methyltransferase ModM3

**DOI:** 10.1128/MRA.01559-19

**Published:** 2020-04-02

**Authors:** Luke V. Blakeway, Aimee Tan, Ian R. Peak, John M. Atack, Kate L. Seib

**Affiliations:** a Institute for Glycomics, Griffith University, Gold Coast, Queensland, Australia; b School of Medical Science, Griffith University, Gold Coast, Queensland, Australia; University of Maryland School of Medicine

## Abstract

Moraxella catarrhalis is a leading bacterial cause of otitis media and exacerbations of chronic obstructive pulmonary disease. Here, we announce a transcriptome RNA sequencing data set detailing global gene expression in two M. catarrhalis CCRI-195ME variants with expression of the DNA methyltransferase ModM3 phase varied either on or off.

## ANNOUNCEMENT

Moraxella catarrhalis is a leading bacterial cause of otitis media in children and exacerbations of chronic obstructive pulmonary disease in adults (1). M. catarrhalis contains a type III *N*^6^-adenine DNA methyltransferase, ModM, that exhibits phase-variable expression mediated by a 5′-(CAAC)*_n_*-3′ tetranucleotide repeat tract in the open reading frame of the gene (2, 3). Six *modM* alleles (*modM1* to *modM6*) that vary in their target recognition domains have been identified and are expected to methylate different target sequences (4). We showed previously that on/off switching of ModM2 epigenetically regulates the expression of a phase-variable regulon (phasevarion) containing 34 genes, including genes important for colonization and infection (2). Although *modM2* is the most frequently occurring allele, *modM3* is overrepresented in strains isolated from the middle ears of children during episodes of otitis media (2, 4). Here, we present a transcriptome RNA sequencing (RNA-seq) data set describing the effect of ModM3 phase variations on global gene expression in the otitis media isolate M. catarrhalis CCRI-195ME.

M. catarrhalis CCRI-195ME ModM3 on and off variants were isolated as described previously (5, 6). Triplicate biological replicates of each variant were grown to mid-log phase in brain heart infusion broth at 37°C with orbital shaking at 200 rpm. Total RNA was extracted using the RNeasy minikit (Qiagen), and residual DNA was digested using the RNase-free DNase set (Qiagen). RNA-seq was performed by the Australian Genome Research Facility (Melbourne, VIC, Australia). rRNA was depleted using the Ribo-Zero Gold kit (Illumina). cDNA was synthesized using SuperScript II reverse transcriptase (Invitrogen), and libraries were assessed for correct sizing using a Bioanalyzer DNA 1000 chip (Agilent). Libraries were quantified with quantitative PCR and normalized to 2 nM. Pooling and clustering of libraries were performed using the Illumina cBot system with TruSeq PE cluster kit v3 reagents; 150-bp paired-end sequencing was performed on the Illumina MiSeq system with TruSeq SBS kit v3 reagents. Reads were mapped to the M. catarrhalis CCRI-195ME genome (GenBank accession number ASM208012v1) using STAR aligner v2.5.3a (7) (https://github.com/alexdobin/STAR). The Subread package utility featureCounts v1.4.6-p5 (8) (http://subread.sourceforge.net) for R v3.5.0 was used to quantitate the number of reads mapped to each gene. Default parameters were used in all programs except where otherwise noted. Sequencing yielded 15,067,808 reads in total, and the summary of sequencing reads and mapping results can be found in Table 1. This data set can be used to examine the suite of genes that are epigenetically regulated by phase variation of the ModM3 methyltransferase and will be an important resource to determine the stably expressed antigenic repertoire of M. catarrhalis, facilitating the design of an M. catarrhalis vaccine.

**TABLE 1:** Summary of sequencing reads

Sample	No. of reads	% of reads mapped to reference genome	% of reads mapped to rRNA	SRA accession no.
ModM3 ON1	2,304,914	96.45	0.00	SRX7141657
ModM3 ON2	2,180,113	90.48	0.00	SRX7141658
ModM3 ON3	2,929,573	96.02	0.00	SRX7141659
ModM3 OFF1	2,480,409	96.27	0.00	SRX7141660
ModM3 OFF2	2,764,121	96.02	0.00	SRX7141661
ModM3 OFF3	2,408,678	96.08	0.00	SRX7141662

### Data availability.

Data were deposited in GEO under data set accession number GSE140417.
